# Possible additional value of 18FDG-PET in managing pancreas intraductal papillary mucinous neoplasms: Preliminary results

**DOI:** 10.1186/1756-9966-27-10

**Published:** 2008-06-10

**Authors:** Gian Luca Baiocchi, Nazario Portolani, Francesco Bertagna, Federico Gheza, Claudio Pizzocaro, Raffaele Giubbini, Stefano M Giulini

**Affiliations:** 1 Department of Medical and Surgical Sciences, Surgical Clinic, University of Brescia, Brescia, Italy; 2 Nuclear Medicine Service, Brescia Civil Hospital, Brescia, Italy

## Abstract

Although some clinical and radiological features may predict malignancy presence in intraductal papillary mucinous pancreas neoplasms, preoperative diagnosis remains difficult. In this study we present 7 patients with Intraductal Papillary Mucinous Neoplasm (IPMN) studied both with 18FDG-PET and magnetic resonance cholangiopancreatography (MRCP). A focal hypermetabolism was documented in 2 patients (the standardized uptake value in the neoplastic foci was 6.7 and 9), while absence of FDG uptake in the neoplasm area was recorded in the remaining 5 cases. Mean follow-up was 27 months (range 21–34). The final judgement was benign IPMN in 5 cases and malignant IPMN in 2. PET scan always correctly predicted the presence or absence of malignancy, while MRCP failed to detect malignancy in 3/7 cases. In conclusion, this preliminary experience suggests that 18FDG-PET may prove useful for malignancy detection in IPMN, improving differential diagnosis with benign intraductal papillary growth by functional data.

## Background

Cystic pancreas tumours are observed with increasing frequency in asymptomatic patients as incidental findings during US or CT abdominal imaging. Pancreatic resection, the only available therapy, may represent an over-treatment due to the low malignant potential of more than half of these lesions [1]. Within the whole group of cystic pancreas tumours, preoperative imaging, including magnetic resonance cholangiopancreatography (MRCP) and endoscopic ultrasound, usually allows three main lesions to be differentiated: serous adenoma, mucinous tumour and IPMN (intraductal papillary mucinous neoplasm) [2]. While in the two former tumours the indications are well recognised (radiological follow-up for serous and resection for mucinous neoplasms), IPMN still represents a more critical field, potentially bearing adenoma, in situ carcinoma or invasive carcinoma; these lesions cannot be differentiated with sufficient accuracy by currently available imaging techniques.

In the search for a diagnostic tool improving malignant IPMN preoperative detection accuracy, 18FDG-PET was poorly investigated in the published series. This paper's aim is to discuss the potential of this functional imaging technique in managing IMPN, based on a preliminary monocentric experience.

## Methods

In the period 2003–2005, 28 patients with a conclusive diagnosis of IPMN were observed in the Surgical Clinic of Brescia University. The radiological workup included magnetic resonance cholangiopancreatography (MRCP) in 26 patients and CT scan in 23 patients. Endoscopic ultrasonography was performed in 13 patients (in 6 cases with fine needle aspiration of the cystic contents). MRCP was conducted with noncontrast, T2-weighted, fat-suppressed, HASTE sequences, T1-weighted, fat-suppressed WIBE sequences, and dynamic gadolinium administration; postcontrast MR images were acquired on transverse, coronal and axial planes. 16-row multidetector CT scan was performed with water gastric filling and endovenous contrast medium injection; reconstruction along the course of the main pancreatic duct was possible using multiplanar reformatted images and 3-dimensional representations.

In 7 cases, 18FDG-PET/CT was performed. All the patients had fasted for at least 6 hours beforehand, had glucose levels below 150 mg/dl, and good hydratation. 5.5 MBq/Kg were injected intravenously. 2D mode OS-EM imaging (with septa) was acquired 60 minutes after injection on a Discovery ST PET/CT tomograph (GE^®^) using CT for attenuation correction (characteristics: 80 mA, 120 Kv without contrast; CT slice thickness of 3.75 mm to approximate the PET slice width; reconstructed slice interval of 3.27 mm to match the PET slice spacing; 4 minutes per bed-PET-step of 15 cm). The PET images were analyzed visually and semi-quantitatively using the standardized uptake value (SUVbw, g/ml). The reconstruction was performed in a 128 × 128 matrix and 60 cm FOV (field of view). Transaxial, coronal, and sagittal sections were obtained for visual analysis, performed according to a colour scale. All patients gave written consensus to perform the exam.

Surgical resection was proposed, according to the International Consensus Guidelines [3], for all main duct type IPMNs, and for branch duct type IPMNs larger than 3 cm or symptomatic or with mural nodules at MRCP. The final diagnosis of benign or malignant IPMN was deduced from the pathological report in the resected patients and from the clinical and radiological follow-up in the observed patients, in accordance with the criteria adopted in recently published series [4-6].

## Results

**Figure 1:**
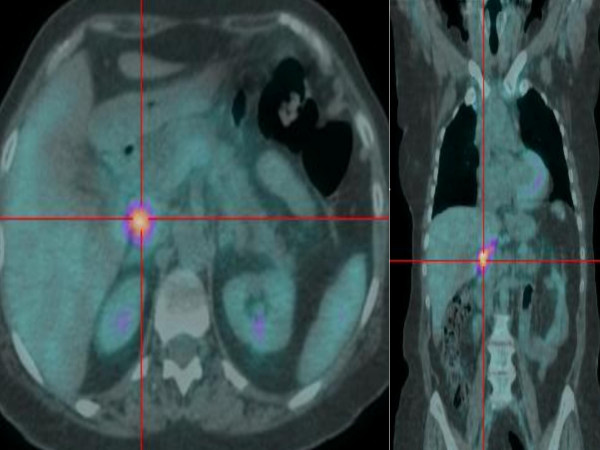
**PET scan of patient N. 2, showing a focal hyperintensity signal at pancreatic head level;** the magnetic resonance cholagiopancreatography (MRCP) showed the presence of a cystic dilatation of the whole Wirsung duct. Total pancreasectomy was performed, with a final diagnosis of in situ carcinoma within the cephalic Wirsung duct.

An intense focal hyper metabolism was documented by 18FDG-PET in 2 patients (SUV in the neoplastic foci was 6.7 and 9 respectively); 18FDG-PET images showed complete absence of FDG uptake in the neoplasm area in the remaining 5 cases (Table 1). In the 2 PET positive cases, MRCP also presented morphological aspects indicative of potential malignancy, as the main duct was involved in both and a nodal metastasis was suspected in one. The first patient, an 83 year-old man with severe chronic respiratory distress, was not operated owing to high surgical risk; percutaneous jaundice palliation was performed, and the patient died from the tumour 5 months later. The second patient was a 67 year-old female submitted to total pancreasectomy; histological examination demonstrated in situ carcinoma, located in the cephalic region where the PET scan showed a focal hyperintensity signal (Fig. 1). Out of the 5 PET negative patients, 3 were MRCP positive for potential malignancy, because of main duct involvement (1 case) and mural nodules located inside a branch duct type cyst larger than 3 cm (2 cases), one of which increasing in size in serial examinations. Two of these PET negative/MRCP positive patients were resected with definitive histological diagnosis of benign IPMN, while the third patient, an 81 year-old woman candidate to pancreatico-duodenectomy, was excluded from surgery owing to the high surgical risk and is alive and with an unmodified cyst at the 26 month control. The remaining two patients were both PET and MRCP negative for malignancy; they were observed, and their lesions were found to be stable in size and structure by MRCP controls, respectively after 21 and 34 months follow-up.

**Table 1:** Data summary of 7 patients with IPMN undergoing magnetic resonance cholagiopancreatography (MRCP) and 18FDG-PET scan in the preoperative workup.

Pt	**Data indicative of malignancy**		**Therapy**	**Final diagnosis**
	**MRCP**	**PET**		

1	POSITIVE	POSITIVE	Follow-up*	Malignant
2	POSITIVE	POSITIVE	Total pancreatectomy	Malignant
3	POSITIVE	NEGATIVE	Distal pancreatectomy	Benign
4	POSITIVE	NEGATIVE	Distal pancreatectomy	Benign
5	POSITIVE	NEGATIVE	Follow-up** (26 months)	Benign
6	NEGATIVE	NEGATIVE	Follow-up (21 months)	Benign
7	NEGATIVE	NEGATIVE	Follow-up (34 months)	Benign

* not operated for high surgical risk, dead for disease after 5 months

** not operated for high surgical risk

On the basis of the final evaluation obtained by histopathology or follow-up, in the 7 cases studied by MRCP and PET scan, MRCP gave 3 false out of 5 responses positive for malignancy with no false negatives, while the PET diagnosis was always correct in confirming or excluding malignancy.

## Discussion

The IPMN therapeutic strategy mainly depends on the suspicion of malignancy emerging from the preoperative workup. Although some radiological features have been described that may indicate a definite risk – all main duct tumours and branch duct tumours larger than 3 cm, symptomatic or harbouring parietal nodules [3] – no clinical, biological, biochemical and radiological factors can be considered sufficiently accurate to confirm or exclude malignant component presence in cyst walls [7-10], frequently leading to an aggressive approach to lesions which finally prove to be absolutely benign. Considering that pancreatic resection is a very invasive operation, with mortality of 5–10% and morbidity of 20–40%, and that in most IPMNs the only therapeutic procedure able to remove the disease entirely is total pancreasectomy, a diagnostic tool improving malignancy diagnosis specificity may represent substantial progress in managing these lesions. The accuracy of CT scan is very low in this field, while MRCP is the gold standard diagnostic procedure, due to its ability to demonstrate the Wirsung duct anatomy and its connections to branch-sided cysts, and to exclude the presence of parietal nodules or filling defects. In the present series, considering the entire cohort of patients, MRCP proved to be better than CT both for a correct diagnosis of the pancreatic cystic neoplasm as IPMN (100% versus 73.9%) and for the recognition of patients at high risk of malignancy (sensibility, specificity, positive and negative predictive value were respectively 100%, 75%, 54.4% and 100% for MRCP and 40% 88.8%, 50% and 84.2% for CT).

Positron emission tomography (18FDG-PET) is a functional imaging technique that has been proposed as a valuable tool for diagnosing and staging different malignancies, including pancreatic adenocarcinoma [11]. Some published experiences have reported low 18FDG-PET sensitivity in detecting malignant tissue presence in cystic lesions: 57% in the Mansour study including 68 patients with pancreatic cystic tumours [4], and 59% in 22 patients with mucinous carcinoma from a variety of organs, including 2 pancreatic tumours, presented by Berger and Coll. [5]; these figures were considered as inadequate by the Authors, concluding that no therapeutic decision could be based on PET scan. On the contrary, Sperti and Coll. reported a series of 56 cystic pancreas neoplasms, in which 18FDG-PET sensitivity, specificity, positive and negative predictive values were respectively 94%, 97%, 94%, and 97% [6]. From the cited papers, including all the different types of pancreatic cystic tumours, it is impossible to enucleate the data concerning the IPMN subgroup. Five and 17 IPMNs were respectively present in the Memorial Sloan Kettering Cancer Centre study [4] and from Italy [6]. In these papers, three cases of IPMNs with in situ carcinoma and one case of invasive papillary carcinoma were correctly marked positive at PET scan, but one IPMN case with in situ carcinoma without metabolic activity at PET was described by Mansour, while no false negative PET scans were reported by Sperti. To the best of our knowledge no further paper specifically focused on this topic has been published, apart from a report of 2 cases of IPMNs studied by PET described by Yoshioka in 2003 [12].

In our patients the 18FDG-PET results always agreed with the final malignancy: in the 4 patients with definitive diagnosis (3 histological examination and 1 disease-related death), PET added specificity to MRCP, correctly excluding malignancy in the 2 benign cases, which presented aspects considered indicative of malignancy at MRCP, and demonstrated good sensitivity, showing high FDG uptake areas in the two malignant lesions. The remaining 3 cases not submitted to resection, classified as benign on the basis of the uneventful follow-up in accordance with the criteria adopted by the previously cited studies [4-6], were correctly defined negative at PET, confirming PET accuracy in excluding malignancy. PET scan was therefore always accurate in the 7 cases of this series, correctly defining the nature of the 5 benign lesions, as well as of the 2 malignant ones. The PET positivity of an situ carcinoma in 1 patient is particularly interesting, confirming the 3 similar cases previously described [4,6], and means that even a small amount of tissue with increased metabolic activity, in an initial phase of the cancerization process, may be detected. By analysing metabolic activity within the wall of a cystic lesion, PET scan offers the possibility of investigating the nature of small mural nodules, morphologically demonstrated by MRCP by functional data, so discriminating benign and malignant lesions.

## Conclusion

PET scan, combined with MRCP, may thus add specificity to a preoperative study based on MRCP alone, preventing unnecessary pancreatic resections. Its value in managing IPMN merits further investigation.

## Authors' contributions

GB conceived of the study and drafted the manuscript, FB, CP and RG performed the nuclear medicine examinations, FG, NP and SMG participated in the design of the study. All authors read and approved the final manuscript.
